# The Reliability of the Tracheoesophageal Groove and the Ligament of Berry as Landmarks for Identifying the Recurrent Laryngeal Nerve: A Cadaveric Study and Meta-Analysis

**DOI:** 10.1155/2017/4357591

**Published:** 2017-02-08

**Authors:** Brandon Michael Henry, Beatrice Sanna, Matthew J. Graves, Silvia Sanna, Jens Vikse, Iwona M. Tomaszewska, R. Shane Tubbs, Krzysztof A. Tomaszewski

**Affiliations:** ^1^International Evidence-Based Anatomy Working Group, Krakow, Poland; ^2^Department of Anatomy, Jagiellonian University Medical College, Krakow, Poland; ^3^Faculty of Medicine and Surgery, University of Cagliari, Sardinia, Italy; ^4^Department of Surgical Sciences, University of Cagliari, Sardinia, Italy; ^5^Division of Medicine, Stavanger University Hospital, Stavanger, Norway; ^6^Department of Medical Education, Jagiellonian University Medical College, Krakow, Poland; ^7^Seattle Science Foundation, Seattle, WA, USA

## Abstract

*Purpose*. The aim of this meta-analysis was to provide a comprehensive evidence-based assessment, supplemented by cadaveric dissections, of the value of using the Ligament of Berry and Tracheoesophageal Groove as anatomical landmarks for identifying the Recurrent Laryngeal Nerve.* Methods*. Seven major databases were searched to identify studies for inclusion. Eligibility was judged by two reviewers. Suitable studies were identified and extracted. MetaXL was used for analysis. All pooled prevalence rates were calculated using a random effects model. Heterogeneity among included studies was assessed using the Chi^2^ test and the *I*^2^ statistic.* Results*. Sixteen studies (*n* = 2,470 nerves), including original cadaveric data, were analyzed for the BL/RLN relationship. The RLN was most often located superficial to the BL with a pooled prevalence estimate of 78.2% of nerves, followed by deep to the BL in 14.8%. Twenty-three studies (*n* = 5,970 nerves) examined the RLN/TEG relationship. The RLN was located inside the TEG in 63.7% (95% CI: 55.3–77.7) of sides.* Conclusions*. Both the BL and TEG are landmarks that can help surgeons provide patients with complication-free procedures. Our analysis showed that the BL is a more consistent anatomical landmark than the TEG, but it is necessary to use both to prevent iatrogenic RLN injuries during thyroidectomies.

## 1. Introduction

**Figure 1:**
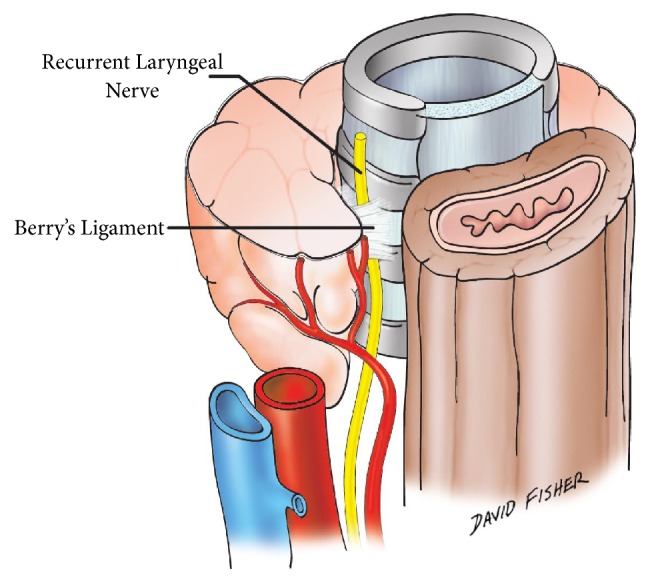
Anatomical location of the Berry Ligament.

Surgeons use various techniques to identify the Recurrent Laryngeal Nerve (RLN) during operative procedures on the neck. These range in palpation, direct inspection, intraoperative nerve monitoring, and anatomical landmarks such as the suspensory ligament of the thyroid gland (Ligament of Berry, BL) and the Tracheoesophageal Groove (TEG). The BL is a fibrous structure that anchors the thyroid gland to the first three rings of the tracheal cartilage [1, 2] (Figure 1). It can help to identify the RLN during surgical procedures, but this has yet to be widely accepted and implemented as standard practice [3]. The TEG, the sulcus formed by the abutment of the trachea anteriorly and esophagus posteriorly, is also useful for identifying the RLN [4].

The RLN is the structure most at risk for iatrogenic injury during procedures on the anterior neck, particularly thyroidectomy [5]. The most common site of injury to the RLN is near the BL, where the nerves penetrate into the larynx [3, 6]. A reliable landmark and method for identifying the RLN is necessary to prevent postoperative complications such as hoarseness and vocal cord paralysis. The location of the RLN with respect to the BL has varied widely in previous reports, ranging from a piercing pattern in 0% of cases in Berlin and Lahey (1929), Çakir et al. (2006), Freschi et al. (1994), Hunt et al. (1968), Leow and Webb (1998), and Sasou et al. (1998) [2, 7–11] to 31.6% in Pradeep et al. (2012) [12]. A more complete understanding of the frequency with which nerves penetrate the BL will reduce complications, as traction-related injuries are associated with RLNs taking a piercing course [6, 10]. The TEG can also be useful for identifying the RLN and offers a fairly safe haven for the RLN as it ascends towards the larynx [13]. An unaware surgeon could overlook a nerve coursing in the TEG because its course is somewhat concealed, so the TEG needs to be assessed for the presence of the RLN [13].

Reports of the location and relationship of the RLN to the BL and TEG have varied widely. The reported presence of an RLN coursing within the TEG has ranged from 24.9% to 100% [14, 15]. The RLN's relationship to the BL seems to be even more variable, with the nerve coursing superficially to the ligament in anywhere between 0% and 100% of cases [2, 7, 8, 11, 16]. The high variability of the data published to date allows no firm assessment to be made of the value of the BL or TEG as landmarks for neural identification.

The high rate of RLN injury, and the need to protect the structure during surgery, has motivated extensive research during recent years. However, a reliable and uniform method for isolating and safeguarding the nerve has yet to be formulated. Our intention is to provide a comprehensive evidence-based assessment, supplemented by our own cadaveric study, of the anatomical reliability of the BL and TEG as landmarks for identifying the RLN. Reliable landmarks would help to reduce the rate of iatrogenic injury and long term postoperative complications.

## 2. Methods

### 2.1. Dissection

A total of 36 formalin-fixed cadavers were dissected bilaterally at the Department of Anatomy, Jagiellonian University Medical College, Krakow, Poland, to investigate the anatomical relationship of the RLN to the BL and TEG. A midline vertical incision was made starting from the mentum and continuing inferiorly to the sternal notch. The dissection was continued through the subcutaneous adipose tissue. Subplatysmal flaps were then raised. Next, the superior and inferior attachments of the sternohyoid and sternothyroid muscles were transected and the dissection was continued until the thyroid gland was visible. All surrounding muscles and adipose and connective tissues were then carefully removed. The RLN was visualized with the aid of medial traction on the trachea. The relationship of the nerve to the BL was recorded (superficial, piercing, or deep). The nerve was then traced inferiorly to the Tracheoesophageal Groove, and the position of the RLN with respect to this groove was recorded (inside or outside: anterior, anterolateral, lateral, and posterior).

### 2.2. Ethics

The research protocol for this study was approved by the Jagiellonian University Medical College Ethics Committee (registry number KBET/319/B/2012). The study was performed in accordance with the ethical standards established in the 1964 Declaration of Helsinki and its later amendments.

### 2.3. Search Strategy

Through August 2016, the major electronic databases PubMed, China National Knowledge Infrastructure (CNKI), ScienceDirect, EMBASE, BIOSIS, SciELO, and Web of Science were searched to identify potential studies for the meta-analysis. No date limits or language restrictions were imposed. The comprehensive PubMed search strategy is presented in Supplement 1 in Supplementary Material available online at https://doi.org/10.1155/2017/4357591. The references of all studies included in the meta-analysis were also searched to identify additional potentially eligible articles. Throughout the meta-analysis, the Preferred Reporting Items for Systematic Reviews and Meta-Analyses (PRISMA) guidelines were strictly followed [17] (Supplement 2). This meta-analysis was prospectively registered in PROSPERO (CRD42015026096).

### 2.4. Study Selection Criteria

Studies were considered eligible for inclusion in the meta-analysis if they (1) reported clear, extractable prevalence data on either the relationship of the RLN to the BL or the relationship of the RLN to the TEG and (2) were cadaveric, intraoperative, or imaging studies. The exclusion criteria included (1) case studies, case reports, conference abstracts, and letters to the editor; (2) studies reporting incomplete data; and (3) studies of patients with trauma to the head and neck region.

Each study was independently assessed for eligibility by at least two reviewers. Any disagreements arising during this assessment were resolved by a consensus among the entire review team, after consultation with the authors of the original study, if possible. Full-text articles in languages not spoken fluently by the reviewers were translated for further eligibility assessment by medical professionals fluent in both English and the original language of the article.

### 2.5. Data Extraction

**Figure 2:**
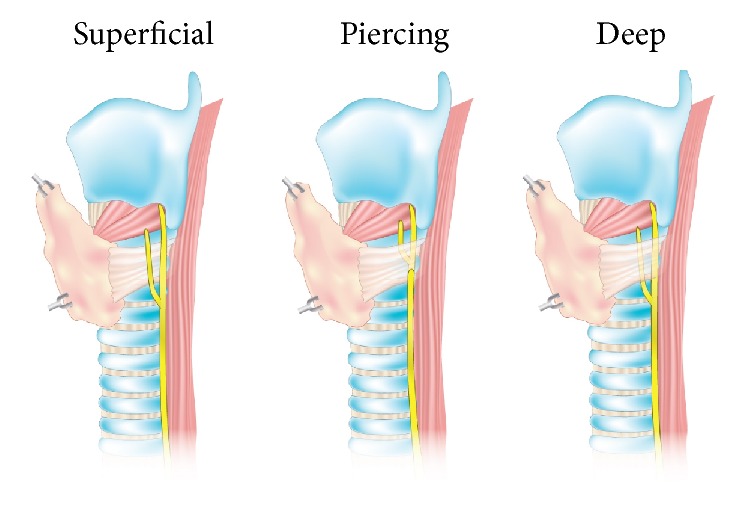
Types of relationship between the Recurrent Laryngeal Nerve and the Berry Ligament.

**Figure 3:**
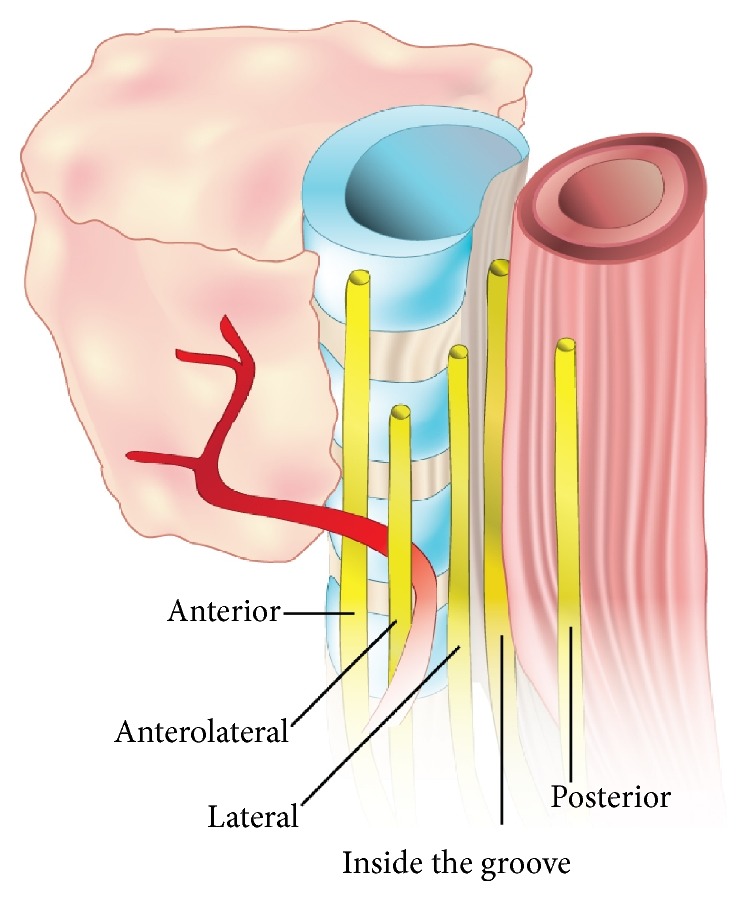
Possible locations of the Recurrent Laryngeal Nerve in relation to the Tracheoesophageal Groove.

Data from the included studies were independently extracted by two reviewers. The extracted data included year, country, type of study, study design, number of nerves, prevalence of the different types of relationships of the RLN to the BL (superficial, piercing, or deep) (Figure 2), prevalence of the different types of relationship of the RLN to the TEG (inside or outside), prevalence of the different positions of the nerve when located outside the TEG with respect to the TEG (anterior, anterolateral, lateral, or posterior) (Figure 3), and symmetry in the aforementioned relationships. In the event of data discrepancies, the review team attempted to contact the authors of the original article by email for clarification.

### 2.6. Statistical Analysis

For the analysis of cadaveric data, prevalence rates and elements of descriptive statistics were used where appropriate. To pool the data into the meta-analysis, pooled prevalence estimates were calculated using MetaXL version 2.0 by EpiGear International Pty. Ltd. (Wilston, Queensland, Australia). A random effects model was used for all meta-analysis calculations. Heterogeneity among the included studies was measured using the Chi^2^ test and the *I*^2^ statistic. For the Chi^2^ test, a Cochran's* Q p* value of <0.10 indicated significant heterogeneity [18]. The results of the *I*^2^ statistic were interpreted as follows: 0–40% might not be important; 30–60% could indicate moderate heterogeneity; 50–90% could indicate substantial heterogeneity; and 75–100% could represent considerable heterogeneity [18].

Subgroup analyses to explore potential sources of heterogeneity were based on type of study (cadaveric versus intraoperative), side (left versus right), and geographical distribution. Confidence intervals of the rates were used to investigate significant differences between subgroups, any overlap between two or more subgroups indicating a lack of statistical significance [19]. When appropriate, for studies with ≥ 100 nerves, sources of heterogeneity were also probed by a sensitivity analysis.

## 3. Results

### 3.1. Cadaveric Study

Among the 36 formalin-fixed cadavers dissected, 16 (44.4%) were men and 20 (55.6%) were women. The mean age was 68.9 ± 11.7 years. The RLN was identified bilaterally in all 36 cadavers (*n* = 72 nerves). In 65 (90.3%) out of the 72 nerves the RLN was located superficial to the BL (Table 1). The relationship was symmetrical in 29 (80.6%) of the 36 cadavers. The RLN was located within the groove in 49 (68.1%) sides and outside it in 23 (31.9%). The relationship was symmetrical in 22 (61.1%) of the 36 cadavers. When the RLN was located outside the groove, it was most commonly found lateral to the TEG (17 nerves, 73.9%) (Table 2).

### 3.2. Study Identification

**Figure 4:**
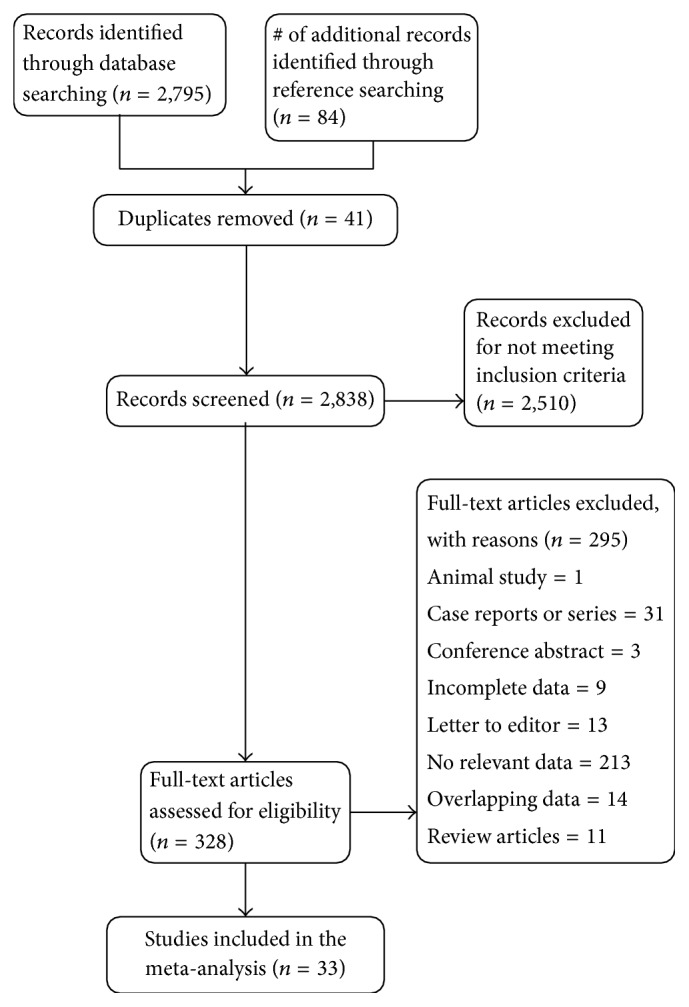
PRISMA flow chart of study identification and inclusion in the meta-analysis.

A summary of the flow of studies through the meta-analysis is presented in Figure 4. A search through the major electronic databases identified 2,795 articles, and 84 more were identified when the references of the included studies were searched. After 41 duplicates had been excluded and 2,838 records screened, 328 articles were further assessed for eligibility by full text. Among these, 295 were excluded, and 33 were included in the meta-analysis.

### 3.3. Meta-Analysis of the Relationship of the Recurrent Laryngeal Nerve to the Berry Ligament

**Figure 5:**
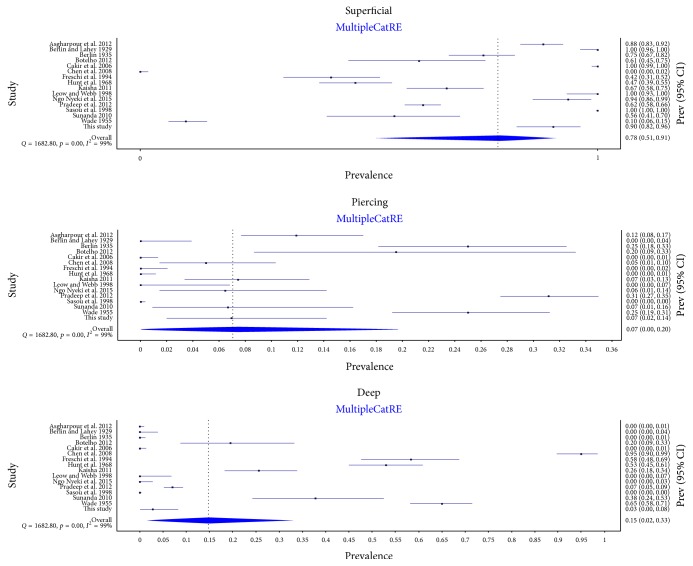
Forest plots for prevalence of Recurrent Laryngeal Nerve relationship pattern with respect to the Berry Ligament.

A total of 16 studies (*n* = 2,470 nerves), including the present one, were included in the analysis of the relationship of the RLN to the BL. The characteristics of the included studies are presented in Table 3. The RLN was most commonly located superficial to the BL with a pooled prevalence estimate of 78.2% (95% CI: 51.5–90.8) of nerves, followed by deep to the BL in 14.8% (95% CI: 0–33.0), and piercing the BL in 7.0% (95% CI: 0–19.6) (*I*^2^ = 99.1%; 95% CI: 98.9–99.2; *p* < 0.001) (Figure 5). The results of the subgroup analyses for types and geographical origins of studies, and a sensitivity analysis, are presented in Table 4. The relationship was reported to be symmetrical in three studies (*n* = 147 subjects). It was found to be symmetrical in 75.5% (95% CI: 64.0–85.4) of subjects (*I*^2^ = 47.5%; 95% CI: 0–84.6; *p* = 0.149).

### 3.4. Meta-Analysis of the Relationship of the Recurrent Laryngeal Nerve to the Tracheoesophageal Groove

**Figure 6:**
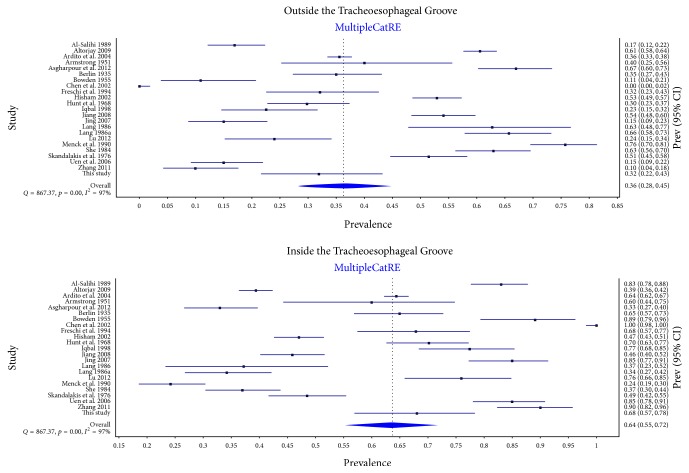
Forest plots for the pooled prevalence of the Recurrent Laryngeal Nerve location with respect to the Tracheoesophageal Groove.

A total of 23 studies (*n* = 5,970 nerves), including the present one, reported data on the relationship of the RLN to the TEG. The characteristics of the included studies are presented in Table 5. The nerve was located inside the groove in 63.7% (95% CI: 55.3–77.7) of sides and outside it in 36.3% (95% CI: 28.3–44.7) (*I*^2^ = 97.4%; 95% CI: 96.8–97.9; *p* < 0.001) (Figure 6). No significant differences were found in the subgroup analysis by type of study, side, or geography, or in the sensitivity analysis (Table 6). In the two studies (*n* = 133 subjects) that reported symmetry data, the relationship of the RLN to the TEG was symmetrical in 44.6% (95% CI: 15.4–75.7) of cases (*I*^2^ = 89.7%; 95% CI: 62.0–97.2; *p* = 0.002).

Ten studies (*n* = 1,268 nerves), including the present one, reported data on the position of the RLN in relation to the TEG when the nerve was located outside the groove. It was located anterior to the TEG in 45.7% (95% CI: 1.1–81.1) of cases and lateral to it in 37.4% (95% CI: 0–72.1). Full results, along with detailed subgroup analysis by type of study, geographical origin, and side, and a subgroup analysis, are presented in Table 7.

## 4. Discussion

Anatomical landmarks such as the BL and TEG come with caveats of which surgeons need to be cognizant before using them in the operating theater.

In our cadaveric dissections, there was a 90.3% prevalence of the RLN coursing superficially to the BL. This coincides with the prevalence rates noted in studies such as those by Asgharpour et al. (2012) and Ngo Nyeki et al. (2015) [1, 3] and with the most common (78.2%) superficial RLN/BL relationship identified in the meta-analysis. The least common type of BL relationship was the piercing pattern, with a 7.0% pooled prevalence estimate. The group with a piercing pattern, although small, needs to be diligently assessed intraoperatively owing to the risk of injuries related to glandular traction [6, 10]. There were no statistically significant differences in subgroup analysis, but there was substantial geographical variability. Most Asian studies reported a lower prevalence of the superficial relationship (59.3%) than their North American, African, and European counterparts, which reported pooled prevalence of 90.7%, 83.4%, and 81.5%, respectively. No differences were noted with respect to study modality (intraoperative versus cadaveric).

Symmetrical RLN patterns with respect to the BL were noted in most cases (80.6%) in our dissections. This information is useful for surgeons performing total thyroidectomies, in that once one nerve is identified, the same location should be assessed first on the contralateral side. The RLN/BL relationship was similarly symmetrical in our meta-analysis: 75.5% of nerves behaved the same on both sides.

Our dissections revealed that the TEG is a somewhat less reliable landmark, only 68.1% of nerves coursing within the groove. This is very similar to the 63.7% pooled prevalence estimate of nerves coursing within the TEG obtained from the meta-analysis. The meta-analysis and cadaveric study differed with regard to the location of the RLN when it was outside the TEG. It was most commonly (73.9%) located lateral to the TEG in our cadavers, but most RLNs outside the TEG (45.7%) were found anteriorly in the meta-analysis. Differences aside, RLNs were found largely, if not exclusively, in locations ranging from anteriorly to laterally. All RLNs in our cadaveric study and 89.1% of cases in our meta-analysis lay within this range. Subgroup analysis of the RLN/TEG relationship revealed no statistically significant differences. Small differences were noted between the cadaveric (34.2%) and intraoperative (39.9%) accounts of those nerves found outside the TEG. We posit that pathologies resulting in these operative procedures, such as large multinodular goiters, slightly alter the physiological location of the RLN. This subanalysis revealed geographical differences, Asian studies generally reporting more RLNs coursing in the TEG (75.9%) than European ones (50.9%). Assessment of RLN/TEG behavior in our cadavers confirmed that the symmetrical pattern of the course (61.1%) was slightly less common than the symmetrical behavior of the RLN with respect to the BL.

The BL has been considered one of the most reliable landmarks in neck surgery [3]. All indications from our data support that claim. The BL should be used in all cases where the RLN needs to be identified. That being said, no structure should be dissected or ligated until the RLN has been identified, because RLN injury is the most common surgical complication of the neck [5, 20]. It is also important that surgeons consider the less common pattern of the RLN piercing the BL. These cases, in which the nerve can be intricately intertwined with the BL, pose a unique hazard in that fibers can easily be severed when the ligament is incised during thyroidectomy [21]. Instances of the RLN piercing the BL are associated with the highest morbidity of nerve palsy [21]. It was highlighted by Serpell in 2010 that the great variability in the relationship of the nerve to the BL could arise from a lack of proper anatomical assessment of the ligament itself [22]. Serpell notes that the BL comprises two fascial layers, and the RLN can be found in the deeper fibrous layer, the “true” BL [22]. This can lead to false reporting of the penetrating pattern of the BL, which has not been directly clarified in studies reporting these variants. Furthermore, Serpell notes that a meticulous dissection of the RLN posterolaterally helps to reduce nerve traction, allowing for safe BL division and reduction in nerve palsies [22].

The TEG is a valuable landmark for identifying the RLN. It provides some shelter, inside which the nerve can be overlooked, but simple palpation of it can provide valuable information about the presence of the RLN [13]. It has also proved useful in laparoscopic procedures of the neck requiring the RLN to be identified [23]. In a series by Chang, the RLN was identified in 100% of laparoscopic thyroid procedures using the TEG [23]. We suggest that an attempt should first be made to identify the RLN in the TEG, and then the course of the nerve should be traced upwards to the BL to confirm the position and structure. This identification can be complicated if there are pathologies, or anatomical variations such as extralaryngeal branching of the RLN. It has been noted that 76.6% of nerves branch prior to entering the larynx [24]. Intraoperative nerve monitoring (IONM) has become more regularly used in recent years although it has yet to be proven superior to concrete anatomical landmarks [5, 25]. That being said, IONM can be beneficial in cases where pathology does not permit these at-risk structures to be adequately visualized and identified. IONM can aid in instances where reoperation is required. It has been noted that transient postoperative complications more than quadruple according to some statistics [26]. The use of the BL and TEG in nerve identification is valuable regardless of whether procedures are of primary or secondary nature and can provide valuable surgical information allowing for reduction of iatrogenic nerve injuries.

Additional research in this area is needed for a number of reasons. Future clinical trials need to be conducted to assess the intraoperative viability of the BL and TEG as landmarks for identifying the RLN, primarily through assessing the prevalence of iatrogenic RLN injury. Establishment of the BL and TEG as truly significant markers for at-risk neural structures requires further prospective studies of high power and sound methodology.

This meta-analysis was limited by several factors: in particular, the studies did not report the location of the RLN with respect to the TEG uniformly. This restricted the overall analysis to the distinction “inside or outside the TEG” rather than a more detailed assessment such as inside, anterior, anterolateral, posterolateral, and posterior. Two separate analyses could be completed on these variables, but they cannot be compared to each other. Moreover, the high heterogeneity among studies persisted despite substantial subgroup analysis, suggesting it could be attributed to intrinsic population variability in prevalence. Further limiting factors included the lack of any quality assessment and risk-of-bias tool for anatomical studies, and a lack of assessment of publication bias, as no statistical measure is currently available for anatomical prevalence meta-analysis. Throughout the study, the original authors were contacted when necessary and available in attempts to resolve discrepancies, provide clarification, and minimize bias.

## 5. Conclusions

The use of anatomical landmarks for identifying structures intraoperatively is valuable in many procedures, including those in the neck. The BL and TEG are both essential for performing complication-free thyroidectomies. The BL proved the more reliable of the two landmarks with 78.2% of RLNs coursing superficially to it. The TEG was slightly less consistent, with 68.1% of nerves found within the groove. The development of a uniform and consistent procedure, such as identifying the RLN in the TEG and tracing it upwards to the BL for confirmation, will help to preclude iatrogenic injuries and avoid complications. The confirmation of reliable landmarks on which to base those procedures is the first step in that process.

## Supplementary Material

Supplement 1: Comprehensive search strategy for PubMed.
Supplement 2: PRISMA 2009 Checklist.

## Figures and Tables

**Table 1:** Cadaveric data on the relationship of the Recurrent Laryngeal Nerve to the Berry Ligament.

	Superficial: # (%)	Piercing: # (%)	Deep: # (%)
Total (*n* = 72)	65 (90.3%)	5 (6.9%)	2 (2.8%)
Right (*n* = 36)	33 (91.7%)	2 (5.6%)	1 (2.8%)
Left (*n* = 36)	32 (88.9%)	3 (8.3%)	1 (2.8%)

**Table 2:** Cadaveric data on the relationship of the Recurrent Laryngeal Nerve to the Tracheoesophageal Groove.

	Inside TEG: # (%)	Outside TEG: # (%)	Outside- anterior: # (%)	Outside- anterolateral: # (%)	Outside- lateral: # (%)	Outside- posterior: # (%)
Total (*n* = 72)	49 (68.1%)	23 (31.9%)	2 (8.7%)	4 (17.4%)	17 (73.9%)	0 (0.0%)
Right (*n* = 36)	26 (72.2%)	10 (27.8%)	2 (20.0%)	1 (10.0%)	7 (70.0%)	0 (0.0%)
Left (*n* = 36)	23 (63.9%)	13 (36.1%)	0 (0.0%)	3 (23.1%)	10 (76.9%)	0 (0.0%)

**Table 3:** Table of studies included in the Recurrent Laryngeal Nerve-Berry Ligament meta-analysis.

Study	Country	Type of study	*n* = (nerves)	Berry's Ligament		
				Superficial (%)	Piercing (%)	Deep (%)
Present study	Poland	C	72	90.3	6.9	2.8
Asgharpour 2012 [3]	England	C	185	88.1	11.9	0
Berlin and Lahey 1929 [7]	USA	I	44	100	0	0
Berlin 1935 [27]	USA	I	140	75.0	25.0	0
Botelho et al. 2012 [28]	Brazil	C	41	61.0	19.5	19.5
Çakir et al. 2006 [8]	Tukey	C	130	100	0	0
Chen et al. 2008 [16]	China	C	100	0	5	95
Freschi et al. 1994 [9]	Italy	I	84	41.7	0	58.3
Hunt et al. 1968 [10]	Australia	C	151	47.0	0	53.0
Kaisha 2011 [29]	Kenya	C	121	67.0	7.4	25.6
Leow and Webb 1998 [11]	England	C	25	100	0	0
Ngo Nyeki et al. 2015 [1]	Cameroon	I	62	93.5	6.5	0
Pradeep et al. 2012 [12]	India	I	584	61.8	31.2	7.0
Sasou et al. 1998 [2]	Japan	I	486	100	0	0
Sunanda et al. 2010 [30]	Sri Lanka	I	45	55.5	6.7	37.8
Wade 1955 [31]	Wales	I	200	10	25	65

**Table 4:** Subgroup analysis for relationship of the Recurrent Laryngeal Nerve to the Berry Ligament.

	Number of studies (number of nerves)	Superficial: % (95% CI)	Piercing: % (95% CI)	Deep: % (95% CI)	*I* ^2^: % (95% CI)^*∗*^
Overall	16 (2470)	78.2 (51.5–90.8)	7.0 (0–19.6)	14.8 (0–33.0)	99.1 (98.9–99.2)
Cadaveric	9 (965)	77.6 (42.0–97.3)	6.8 (0–26.5)	15.5 (0–40.7)	98.8 (98.5–99.1)
Intraoperative	7 (1505)	76.0 (34.9–100)	7.0 (0–32.3)	17.0 (0–49.4)	99.4 (99.2–99.5)
Africa	2 (183)	83.4 (35.9–100)	7.6 (0–44.2)	8.9 (0–47.0)	97.2 (93.0–98.9)
Asia	4 (1215)	59.8 (0–100)	8.4 (0–56.6)	31.8 (0–89.5)	99.6 (99.5–99.7)
Europe	6 (696)	81.5 (35.0–100)	5.3 (0–34.3)	13.2 (0–50.1)	99.1 (98.9–99.3)
North America	2 (184)	90.7 (54.6–100)	9.0 (0–45.4)	0.3 (0–18.1)	96.3 (89.9–98.6)
Sensitivity (≥ 100 nerves)	9 (2097)	70.7 (32.1–92.4)	9.0 (0–30.1)	20.3 (0–46.7)	99.4 (99.3–99.5)

^*∗*^The *p* value of Cochran's *Q* for all analysis was <0.001.

**Table 5:** Table of studies included in the Recurrent Laryngeal Nerve-Tracheoesophageal Groove meta-analysis.

Study	Country	Type of study	*n* = (nerves)	Tracheoesophageal groove	
				Inside (%)	Outside (%)
Present study	Poland	C	72	68.1	31.9
Al-Salihi and Dabbagh 1989 [32]	Iraq	C	212	83.0	17.0
Altorjay et al. 2009 [33]	Hungary	I	1023	39.4	60.6
Ardito et al. 2004 [6]	England	I	1856	64.4	35.6
Armstrong and Hinton 1951 [34]	USA	C	40	60.0	40.0
Asgharpour et al. 2012 [3]	England	C	197	33.0	67.0
Berlin 1935 [27]	USA	I	140	65.0	35.0
Bowden 1955 [35]	England	C	55	89.1	10.9
Chen et al. 2002 [15]	China	C	90	100	0
Freschi et al. 1994 [9]	Italy	I	84	67.9	32.1
Hisham and Lukman 2002 [36]	Malaysia	I	491	47.0	53.0
Hunt et al. 1968 [10]	Australia	C	151	70.2	29.8
Iqbal and Zubair 1998 [37]	Pakistan	I	93	77.4	22.6
Jiang et al. 2008 [38]	China	I	292	45.9	54.1
Jing et al. 2007 [39]	China	C	100	85	15
Lang et al. 1986 [40]	Germany	C	43	37.2	62.8
Lang et al. 1986 [41]	Germany	C	146	34.2	65.8
Lu et al. 2012 [42]	China	I	79	76.0	24.0
Menck et al. 1990 [14]	Germany	C	202	24.3	25.7
She et al. 1984 [43]	China	C	200	37.0	63.0
Skandalakis et al. 1976 [13]	USA	C	204	48.5	51.5
Uen et al. 2006 [20]	Taiwan	C	120	85.0	15.0
Zhang and Cheng 2011 [44]	China	C	80	90.0	10.0

**Table 6:** Subgroup analysis for relationship of the Recurrent Laryngeal Nerve to the Tracheoesophageal Groove.

	Number of studies (number of nerves)	Inside TEG: % (95% CI)	Outside TEG: % (95% CI)	*I* ^2^: % (95% CI)	Cochran's *Q*, *p* value
Overall	23 (5970)	63.7 (55.3–77.7)	36.3 (28.3–44.7)	97.4 (96.8–97.9)	<0.001
Cadaveric	15 (1912)	65.8 (50.7–79.5)	34.2 (20.5–49.3)	97.7 (97.1–98.2)	<0.001
Intraoperative	8 (4058)	60.1 (49.9–70.0)	39.9 (30.0–50.1)	97.0 (95.7–97.9)	<0.001
Asia	10 (1757)	75.9 (60.2–88.9)	24.1 (11.1–39.8)	97.8 (97.0–98.4)	<0.001
Europe	9 (3678)	50.9 (38.0–63.7)	49.1 (36.3–62.0)	97.7 (96.8–98.3)	<0.001
North America	3 (384)	57.5 (45.4–69.1)	42.5 (30.9–54.6)	78.8 (32.3–93.3)	0.009
Right	10 (1597)	62.1 (48.1–75.2)	37.9 (24.8–51.9)	95.3 (93.2–96.8)	<0.001
Left	10 (1554)	68.0 (56.4–78.6)	32.0 (21.4–43.6)	93.4 (21.4–43.6)	<0.001
Sensitivity (≥ 100 nerves)	14 (5334)	54.9 (45.3–64.3)	45.1 (35.7–54.7)	97.6 (97.0–98.2)	<0.001

**Table 7:** Subgroup analysis for position of a Recurrent Laryngeal Nerve located outside the Tracheoesophageal Groove.

	Number of studies (number of nerves)	Anterior: % (95% CI)	Anterolateral: % (95% CI)	Lateral: % (95% CI)	Posterior: % (95% CI)	*I* ^2^: % (95% CI)	Cochran's *Q*, *p* value
Overall	10 (1268)	45.7 (1.1–81.1)	6.0 (0–32.0)	37.4 (0–72.1)	10.9 (0–40.7)	99.4 (99.2–99.5)	<0.001
Cadaveric	7 (531)	47.0 (0–82.0)	7.5 (0–36.6)	26.1 (0–62.6)	19.5 (0–54.8)	98.8 (98.5–99.1)	<0.001
Intraoperative	3 (737)	41.7 (0–100)	2.7 (0–100)	54.2 (0–100)	0.4 (0–100)	99.5 (99.3–99.6)	<0.001
Europe	7 (1098)	23.0 (0–61.6)	3.3 (0–28.6)	59.8 (6.1–93.9)	13.9 (0–49.3)	99.3 (99.1–99.4)	<0.001
North America	3 (170)	85.6 (46.5–100)	10.4 (0–42.1)	0.6 (0–16.5)	3.3 (0–26.2)	95.3 (89.6–97.9)	<0.001
Right	5 (515)	38.0 (0–100)	3.2 (0–47.2)	50.7 (0–100)	8.1 (0–60.7)	99.0 (98.7–99.3)	<0.001
Left	5 (433)	27.1 (0–76.5)	6.3 (0–44.4)	56.7 (0–100)	9.9 (0–52.1)	98.5 (97.8–99.0)	<0.001
Sensitivity (> 100)	4 (1051)	35.7 (0–100)	0.8 (0–100)	42.7 (0–100)	20.8 (0–100)	99.7 (99.6–99.8)	<0.001
